# Supplementary material for: The interactome of intact mitochondria by cross-linking mass spectrometry provides evidence for coexisting respiratory supercomplexes
Source: Mol Cell Proteomics. 2017 Dec 8;17(2):216–32. doi: 10.1074/mcp.RA117.000470 (PMC5795388; doi:10.1074/mcp.RA117.000470)
Supplement: Supplemental Data [file supp_RA117.000470_133922_0_supp_23978_fzffwf.zip › spectra_annotation/mito_DR_spectra_annotation/411-2-10-1-138-1.pdf]

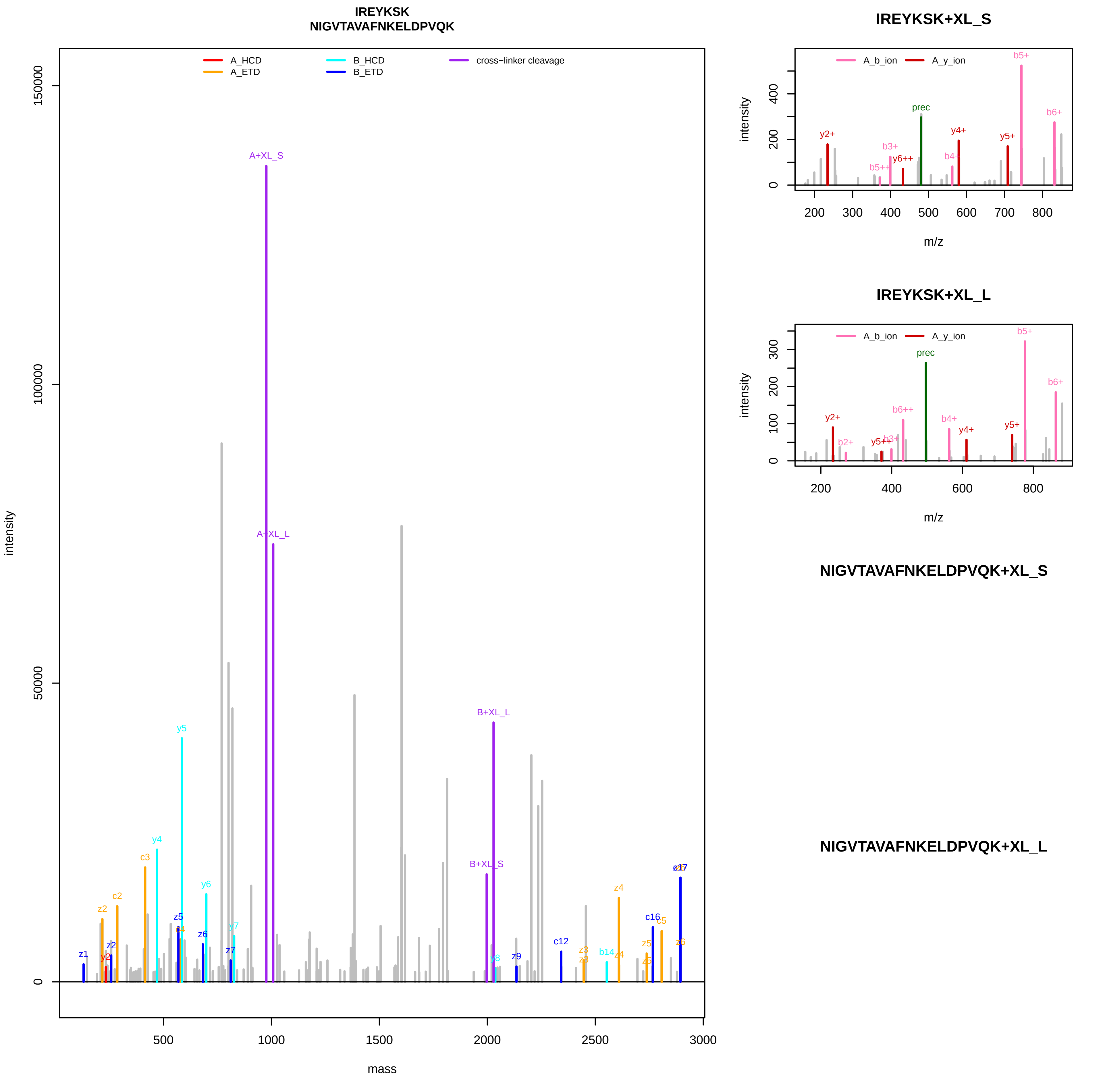
IREYKSK
NIGVTAVAFNKELDPVQK
A_HCD
A_ETD
B_HCD
B_ETD
cross−linker cleavage
A+XL_S
A+XL_L
B+XL_L
B+XL_S
intensity
mass
150000
100000
50000
0
500
1000
1500
2000
2500
3000
z1
z2
y2
c2
c3
y4
y5
z5
y6
z6
y7
z7
y8
z9
c12
z3
b14
z4
z5
c5
c16
z17
z6
IREYKSK+XL_S
A_b_ion
A_y_ion
prec
y2+
b3+
b5++
y6++
b4+
y4+
y5+
b5+
b6+
m/z
IREYKSK+XL_L
b2+
y5++
b6++
NIGVTAVAFNKELDPVQK+XL_S
NIGVTAVAFNKELDPVQK+XL_L
